# Impact of radiofrequency ablation for patients with varicose veins on the budget of the German statutory health insurance system

**DOI:** 10.1186/2191-1991-3-9

**Published:** 2013-04-03

**Authors:** Alexander Kuhlmann, Anne Prenzler, Jan Hacker, J-Matthias Graf von der Schulenburg

**Affiliations:** 1 Leibniz Universität Hannover, Center for Health Economics, Hannover, Germany; 2 Oberender & Partner, Bayreuth, Germany

**Keywords:** Varicose veins, Varices, Costs, Cost analysis, Budget impact, ClosureFast, Stripping, Radiofrequency ablation

## Abstract

**Objectives:**

In contrast to other countries, surgery still represents the common invasive treatment for varicose veins in Germany. However, radiofrequency ablation, e.g. ClosureFast, becomes more and more popular in other countries due to potential better results and reduced side effects. This treatment option may cause less follow-up costs and is a more convenient procedure for patients, which could justify an introduction in the statutory benefits catalogue. Therefore, we aim at calculating the budget impact of a general reimbursement of ClosureFast in Germany.

**Methods:**

To assess the budget impact of including ClosureFast in the German statutory benefits catalogue, we developed a multi-cohort Markov model and compared the costs of a “World with ClosureFast” with a “World without ClosureFast” over a time horizon of five years. To address the uncertainty of input parameters, we conducted three different types of sensitivity analysis (one-way, scenario, probabilistic).

**Results:**

In the Base Case scenario, the introduction of the ClosureFast system for the treatment of varicose veins saves costs of about 19.1 Mio. € over a time horizon of five years in Germany. However, the results scatter in the sensitivity analyses due to limited evidence of some key input parameters.

**Conclusions:**

Results of the budget impact analysis indicate that a general reimbursement of ClosureFast has the potential to be cost-saving in the German Statutory Health Insurance.

## Background

Primary varicose veins are a degenerative disease of the wall of the vein in the superficial vein system of the legs. Various factors (e.g. pregnancy, physical inactivity) affect the characteristics and severity of varices over a lifetime [1]. Primary varicose disease has to be differentiated from less common secondary varicose veins which occur in the deep vein systems to conditions such as deep vein thrombosis, pelvic tumors or arteriovenous fistulae [2]. Besides a variety of symptoms of discomfort in the legs (e.g. itching, heaviness, and aching) [3], varicose veins can cause more severe complications, for instance ulceration, deep main vein insufficiency, varicophlebitis [1,4-6].

The range of reported prevalence data for primary varices is very wide (2-56% in men and 1-60% in women) due to variations in the study population, selection criteria, methods of measurement and disease definition [7-13]. The majority of the literature quotes a study from Callam, which estimates that the prevalence of visible tortuous varicose veins in an unselected Western adult population over the age of 15 years is between 10 and 15% for men and between 20 and 25% for women [7]. According to the “Bonner Venenstudie” [13] 12.4% of men and 15.8% of women had varices without symptoms of chronic vein insufficiency in Germany in 2003. However, the occurrence of varicose veins varies by age [14]. Studies which estimate the incidence of primary varices are rare. Nevertheless, there exists a publication by Mäkivaara et al., who report an incidence rate of 13.5 per 1,000 person years (8.5 for men and 19.2 for women) [15].

Due to the high prevalence the treatment of patients with varicose veins places a substantial financial burden on the health care system. For example, the German Federal Statistical Office estimated health care costs due to varicose veins (ICD-10: I83 Varicose veins of lower extremities) at 790 Mio € in 2008 [16]. Hence, analyses which give a deep insight into costs and benefits of different treatment patterns are important for the German health care sector in order to identify cost effective treatment options.

Several interventional (e.g. surgery, endovenous thermal ablations, sclerotherapy) and non-interventional treatments (e.g. compression therapy) of varicose veins exist and are approved for treatment in Germany. Despite the growing popularity of new minimally invasive endovenous treatments for varicose veins in other countries in the past decade, surgery still represents the standard intervention in Germany [17], since endovenous thermal ablations aren’t covered by the general benefits catalogue of the Statutory Health Insurance (SHI). In contrast to conventional surgery which is often conducted in hospitals with general or regional anaesthesia, the majority of the minimally invasive techniques are performed as office based procedures using tumescent local anaesthesia [18].

Recently, a new endovenous thermal ablation named ClosureFast, which uses radiofrequency techniques to treat varicose veins, was introduced in the USA. This improved version of the ClosurePlus device promises reduced pain, improved Quality of Life (QoL) and a faster recovery after treatment in comparison to surgery. Furthermore, comparable effectiveness parameters are reported [18]. In addition, since ClosureFast is mainly performed in an outpatient setting, treatment costs may be reduced by avoiding costly inpatient procedures. Hence, an inclusion of ClosureFast in the general benefits catalogue of the German SHI should be considered. To explore the potential financial consequences an economic evaluation is necessary.

Besides cost-effectiveness models, budget impact analyses (BIA) are very important in the German setting. The purpose of a BIA is to estimate the affordability of a new health care intervention for health care decision-makers. The results of these models present the impact of an innovation on a national annual health care budget [19]. Such an analysis can be helpful to determine if ClosureFast is an affordable option for the German setting.

Therefore, aim of this study is to examine the budget impact of a general reimbursement of ClosureFast by the SHI in Germany.

Before presenting the model structure, input parameters and analysis procedure in the methods section, we want to give a short overview of the several alternative treatment options.

### Treatment options for varicose veins

Treatment options for varicose veins can be divided into interventional and non-interventional procedures. Conservative, non-interventional treatments such as compression therapies with medical stockings and pantyhose can be used in every stage of the disease. However, they neither remove the varice nor hinder its development. In contrast, non-interventional treatments aim at alleviating the symptoms [1].

The interventional treatments can be categorized in sclerotherapy, endovenious thermal therapy and surgery. Surgical treatment of varicose veins includes high ligation (crossectomy) and saphenous vein stripping, with or without phlebectomy [20] and has been performed since the early 20th century. Until recently, high ligation and stripping was the standard treatment for patients with varicose veins demonstrating improvements in quality of life as well as reductions in symptoms and reoperation rates compared with high ligation and phlebectomies only [21-23]. However, various complications can occur following varicose vein surgery. Even though serious complications such as deep vein thrombosis (1 in 200 patients) or pulmonary embolism (1 in 600 patients) are rare [24], minor complications occur frequently. For instance, reported rates of wound complications including infection, haematoma and abscess formations vary from 3-10% [24,25].

Sclerotherapy techniques aim at inducing endothelial and vein wall damage in a controlled fashion by injecting toxic liquids or foam in the varice. This results in the obliteration of the varicose vein. Sclerotherapy is considered as the gold standard treatment for leg telangiectasias, venulectasias, and reticular veins [26]. A disadvantage is that the treatment of saphenous veins with non-foam sclerosing agents is associated with high recurrent rates [27,28]. However, recently, treatment with ultra-sound guided foam sclerotherapy (UGFS) has shown better results [29-31].

Minimally invasive endovenous thermal treatments include endovenous laser ablation (EVLA) and radiofrequency ablations (RFA). The aim of endovenous thermal ablation is the irreversible obliteration of the varice. For the EVLA procedure, a thin laser fiber is inserted in the vein under duplex ultrasound control and heated by laser energy to cause thermal damage of the vein wall. The ultrasound guided RFA technique uses radiofrequency energy which is directed through a small cathether inserted through a tiny insertion in the vein to heat up the varice and damage its vein wall. In different randomized trials, the first-generation RFA device VNUS ClosurePlus and EVLA showed comparable effectiveness to surgery; however, RFA was associated with reduced pain and improved QoL as well as a faster recovery after treatment [32-42].

In the following section, we will describe the methods used to explore the potential financial consequences of an introduction of ClosureFast in the German SHI.

## Methods

To assess the budget impact of a “World with ClosureFast” compared to a “World without ClosureFast”, a multi-cohort Markov model was developed and programmed in Microsoft Excel 2007.

The evaluation was conducted from the perspective of the SHI in Germany. The publication of the ISPOR task force [43] as well as the manuscript of Nuijten et al. [19] served as guidelines for the preparation of the BIA. Input parameters were derived from a systematic literature research. In addition, two medical experts in the field of varicose veins were contacted to provide expert opinions on present and future market shares of initial and secondary interventional treatment of varicose veins as well as resource use.

### Model structure

**Figure 1:**
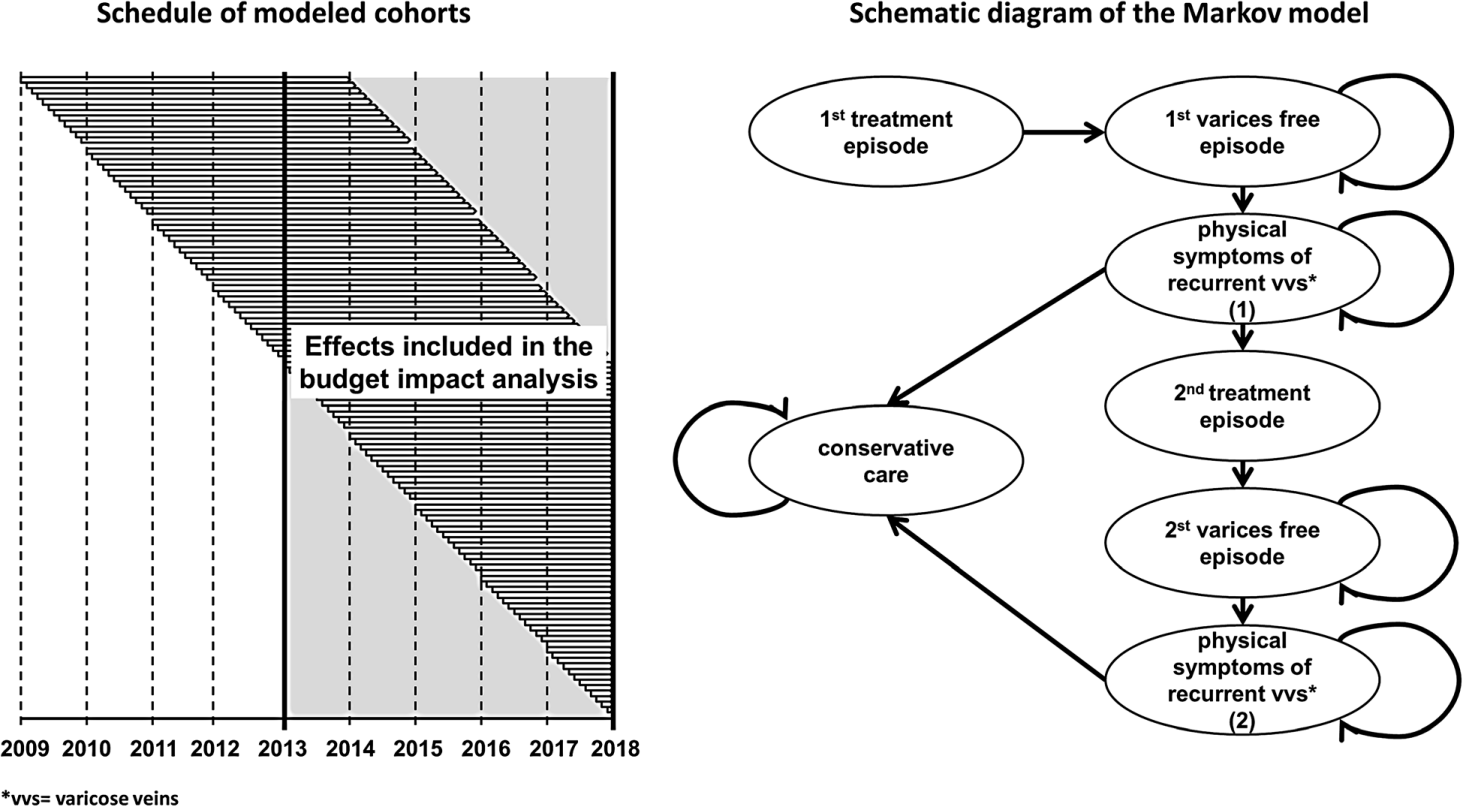
Structure of the multi cohort Markov model.

The basic structure of the model was adapted from a recently published cost-effectiveness model of the National Institute for Health and Clinical Excellence (NICE) [44,45], illustrated in Figure 1. (For the following description of the model structure see NICE [44,45].)

The time horizon of the model was five years with a cycle length of one month. Patients enter the model receiving initial interventional treatment for varicose veins (state: “1st treatment episode”). Following the completion of the treatment episode, patients move to the state “1st varices free episode”, in which they do not require further treatment. They remain in this state until a varicose vein recurs. Hence, they transit to the state “physical symptoms of recurrent varicose veins (1)”. A defined proportion of patients receive a 2nd interventional treatment while the others are treated with conservative care in form of a compression therapy until the end of the model’s time horizon (or death). Patients who undergo a second interventional treatment move on to the state “2nd varices free episode”, where they remain until they experience a 2nd clinical recurrence. In case of a 2nd recurrence, patients only receive conservative care until the end of the model’s time horizon (or death). Transition to death is possible from all states of the model. The annual risk of dying is the same in every health state. The monthly all-cause death probability was calculated on the basis of official life tables [46] (Table 1).

**Table 1:** Key parameters of the model


• treatment episode consists of interventional treatment for every patient and a top-up treatment (additional treatment in case of complications, i.e. vein has not been occluded or obliterated) for a defined proportion of patients (i.e. treated vein is not occluded or obliterated)	
• maximum of two treatment episodes: initial interventional treatment for all patients and a second interventional treatment for a given proportion of patients with recurrent varicose veins	
• no difference of effectiveness between initial and secondary interventional treatment for each procedure	
• top-up treatment is always UGFS and has no influence on recurrence rates	
• constant hazard of recurrence	
• 6 month delay between onset of clinical recurrence and second treatment episode	
• conservative care is given to patients with recurrent varicose veins who do not undergo a second treatment episode as clinical recurrence is not considered clinically meaningful and to all patients with a second recurrence	

Source: NICE [44,45].

To calculate the budget impact, every month, a defined cohort had to enter the model receiving initial interventional treatment according to given market shares. Considering the time horizon of five years (2013–2017), the model included 60 patient cohorts. However, since recurrence rates play an important role in the treatment of recurrent varicose veins (see segment effectiveness) patients with an initial or 2nd interventional treatment of varices before 2013 should not be neglected. Hence, we added another 48 cohorts (four years) to the model and followed-up each of these cohorts for five years (see Figure 1).

### Patient cohorts and market shares of treatments for varicose veins

German literature data exists only for the annual number of varicose vein surgeries performed in an inpatient setting. Göckeritz estimates that surgeries still account for about 90% of all interventional treatments for varicose veins in Germany [17]. Under the assumptions that the small number of patients treated with endovenous thermal ablations within the SHI can be neglected (expert opinion) and all top-up treatments are UGFS (see Table 1), about 95% of all interventional non-top-up treatments have to be surgical and 5% sclerosing procedures. Furthermore, about 60% of varicose vein surgeries are undertaken in an outpatient setting according to experts’ estimates.

If ClosureFast is introduced in the SHI benefit catalogue, the market shares will shift – in particular at the expense of surgery market shares. On the basis of expert opinion, we assume that ClosureFast accounts for 10% of all procedures in the year of its introduction and has an annual market growth of five percentage points each following year (Base Case assumption). The increase of the use of ClosureFast is assumed to reduce the market share of surgeries equally. The medical experts do not assume a reimbursement of EVLA by the SHI in the near future; therefore, EVLA treatment was not included in the budget impact analysis (Table 2).

**Table 2:** Assumed market shares of interventional treatments for varicose veins in Germany*

**Market shares**	**2013**	**2014**	**2015**	**2016**	**2017**
*World without ClosureFast (1st and 2nd treatment)*					
Surgery	0.95	0.95	0.95	0.95	0.95
UGFS	0.05	0.05	0.05	0.05	0.05
*World with ClosureFast (1st and 2nd treatment)*					
Surgery	0.85	0.80	0.75	0.70	0.65
ClosureFast	0.10	0.15	0.20	0.25	0.30
UGFS	0.05	0.05	0.05	0.05	0.05

* Excluding top-up treatment.

In Germany, inpatient varicose vein surgeries are coded as OPS 5–385 within the G-DRG system. Based on the numbers of coded surgical procedures which were derived from the German Federal Statistical Office [47] as well as the above stated expert opinions on market shares, we calculated the size of the monthly patient cohorts. Table 3 shows the different steps of the calculation process in detail.

**Table 3:** Calculation of monthly patient cohorts

	Annual inpatient surgeries OPS 5-385*	**Annual inpatient surgeries of SHI patients**	**Annual outpatient surgeries of SHI patients**	**Annual total surgeries of SHI patients**	**Annual total initial surgeries of SHI patients**	**Annual total initial surgeries + UGFS of SHI patients**	**Monthly total initial surgeries + UGFS of SHI patients**
2009	147,445	125,328	187,992	313,321	250,657	263,849	21,987
2010	146,279	124,337	186,506	310,843	248,674	261,762	21,814
2011	146,351	124,398	186,598	310,996	248,797	261,891	21,824
2012-2017	146,000^1^	124,100	186,150	310,250	248,200	261,263	21,772
Calculation	I	II = I • 0.85 (85% of patients in SHI)^2^	III = II • 1.5 (outpatient/inpatient ratio)^3^	IV = II + III (inpatient + outpatient)	V = IV • 0.8 (initial procedures account for 80% of total procedures)^4^	VI = V • 1/0.95 (surgeries account for 95% of all non-top-up treatments)^5^	VII = VI • 1/12

^1^ Assumption.

^2^ German Federal Statistical Office [48].

^3^ Expert opinion.

^4^[49-53].

^5^ Calculated based on Göckeritz [17], NICE [44] and expert opinion.

* The annual inpatient surgeries do not reflect the number of treated patients per year but the number of inpatient reimbursed procedures. The annual number of patients treated in an inpatient setting is lower [54].

### Effectiveness and adverse events

The effectiveness of the different treatment procedures was modeled via recurrence data. “Success” of interventional treatment was not considered in the model, as patients receive top-up treatments (additional treatment in case of complications, i.e. vein has not been occluded or obliterated) until the treatment episode is complete [44,45] (NICE).

A recurrence describes varicose veins which occur in the same area of the treated varice, independently of the type of the initial treatment. Noppeney et al. distinguish between three causes of recurrent varices: technical failure of the initial treatment, neovascularisation and progression of the venous disease [55]. About 20% of varicose surgeries are attributed to recurrences [49-53]. Differences in the initial treatment, the method of measuring recurrences and duration of follow-up make a comparison of recurrence rates difficult [56]. After surgical treatment of varicose veins and a follow-up period of three to eleven years, recurrence rates of 26-62% have been reported [57,58]. In addition, data indicates that the rate of recurrences increases over time [59].

To gather data on recurrence rates as well as adverse events of all relevant treatment options for varicose veins, we performed a structured literature search in 44 databases using the database search tool of the German Institute of Medical Documentation und Information (DIMDI). Additionally, we conducted a hand research. The search strategy targeted meta-analyses of RCTs for surgery and UGFS as well as RCTs for ClosureFast.

The systematic literature review identified five meta-analyses [32,44,60-62] which compared recurrence data as well as adverse events of RFA and surgery. Two of these studies [32,44] included a comparison between foam sclerotherapy and surgery. Only one RCT was found which reported recurrence rates as well as adverse events of ClosureFast in comparison with surgery and UGFS [18]. The study was also included in two of the identified meta-analyses [44,62]. In total, six RCTs [18,36,63-66] presenting relevant data on recurrence rates have been identified via the five meta-analyses. Table 7 of the Appendix presents an overview of the reported recurrence rates. Only Belcora et al. found a statistically significant difference in recurrences [63].

In the Base Case analysis, we used the effectiveness data reported by NICE. Using a network meta-analysis approach, the authors estimated a one-month recurrence probability of 0.008331 for surgery, 0.005833 for endovenous thermal ablations and 0.009141 for UGFS. However, only a combined probability for RFA and EVLA was calculated [44]. Since none of the other identified RCTs and meta-analyses found a statistically significant difference between RFA and surgery, we made the conservative assumption that ClosureFast had the same effectiveness as surgery.

Adverse events were not included in the analysis. The complications reported in different trials varied a lot and different measurement methods were used. According to a multidisciplinary Guideline Development Group, which currently develops the NICE guidelines for the diagnosis and management of varicose veins, adverse event profiles of the different interventions are similar to the extent that they can be neglected in health economic models [44,45].

### Resource use and cost data

Resource and cost data regarding the treatment of varicose veins as well as the resulting side effects are only available in the literature to a limited extent. Therefore, we have identified relevant treatment procedures and resource usage via expert opinion and official handbooks. Hence, we have evaluated the resource use from the perspective of the SHI in Germany, taking also into consideration patient co-payments as well as discounts for medications given by the manufacturer and pharmacies as required by legal obligations in Germany. For this, the current German recommendations [67,68] for the valuation of resource usage were applied to evaluate the costs from the perspective of the SHI. In the Base Case analysis, we assumed that costs of interventional treatments in 2012 and costs of compression therapy in 2011 (no newer data currently available) would stay stable over time. Considering the increasing costs of inpatient treatment of varicose veins in recent years, this is a conservative assumption, since rising prices of inpatient procedures would have a greater effect on overall surgery costs due to its higher inpatient ratio compared with ClosureFast. However, price assumptions were varied in sensitivity analyses, e.g. by using the mean annual price inflation rate of varice treatments between 2005 and 2012, in order to take the uncertainty regarding future price developments into account. The historic development of surgery and UGFS prices are shown in Table 8 of the Appendix.

In Germany, inpatient stays are reimbursed via lump sums (diagnosis related groups, DRG). In these payments, in general all expenses of the hospital, incl. medication costs, are included. Relevant DRG were identified via the German DRG-Catalogue [69] using the official German DRG Definition Handbook from 2012 [70] and evaluated from the perspective of the German SHI [67-70].

Regarding the outpatient setting, we applied the official German Uniform Valuation Scheme (EBM) [71] and identified relevant OPS-codes for outpatient operations for surgery as well as physician visits. Again, the above mentioned recommendations for the evaluation were used [67,68]. For general outpatient physician visits of SHI-insured persons, it is important to mention that the doctors are reimbursed via lump sum payments per quarter, independent from the number of visits of an individual patient per quarter.

The proportion of patients treated in an outpatient and inpatient setting, respectively, was identified via expert opinion, since no official figures or literature data was available. Due to the uncertainty of the proportions, extensive sensitivity analyses were performed.

Table 4 summarizes the costs and effectiveness as well as the setting of all interventional treatments for varicose veins which were included in the BIA.

**Table 4:** Effectiveness, costs and setting of interventional treatments

**Parameter**	**Value**	**Source**
**Interventional treatment effectiveness**		
*Monthly probability of recurrence after 1st and 2nd treatment*		
Surgery	0.00833	NICE [44]
ClosureFast	0.00833	Assumption
UGFS	0.00914	NICE [44]
*Probability of top-up treatment*		
Surgery	0.05	NICE [44]
ClosureFast	0.05	NICE[44]
UGFS	0.20	NICE [44]
*Probability of receiving 2nd intervention after recurrence*	0.75	NICE [44]
**Treatment setting and costs**		
*Outpatient treatment proportion*		
Surgery	0.60	Assumption
ClosureFast	0.90	Assumption
UGFS	1.00	Assumption
*Inpatient treatment costs*		
Surgery	2,218.02 €	DRG F39b
ClosureFast	2,218.02 €	DRG F39b
*Outpatient treatment costs*		
Surgery	639.45 €	OPS-Code 5–385.70*
ClosureFast	1,100.00 €	Assumption**
UGFS	47.23 €***	EBM-Codes 03111, 30501, 30500
*Monthly costs of conservative compression therapy*	11.45 €	Kemper et al. [72]

* incl. operation and post-operative treatment.

** Average reimbursement in sub contracts between manufacturer and statutory health insurance funds.

*** EBM-Code 03111: 30.84 €, EBM-Code 30500: 16.30 €, EBM-Code 30501: 9.29 €.

### Uncertainty

Various key inputs of the model are based on expert opinions (e.g. outpatient ratio of treatments) or on limited evidence (e.g. recurrence rates of interventional treatments for varicose veins) which may result in high uncertainty of model outcomes. To test the robustness of the model, we conducted three types of sensitivity analyses. First, we performed one-way sensitivity analyses to assess the impact of variations in values of every input parameter on the results. In order to test how the results react to a simultaneous variation of several inputs, we assumed four reasonable alternative scenarios of which two scenarios (ClosureFast + and ClosureFast++) were based on input values more likely to favor a “World with ClosureFast” and two other scenarios based on input values more likely to favor a “World without ClosureFast” (ClosureFast- and ClosureFast--). The alternative scenarios are summarized and described in Table 9 of the Appendix. Furthermore, we calculated the budget impact of the Base Case and the 4 alternative scenarios for three different market uptakes of ClosureFast:

•Base Case market uptake (10 percent points in the year of introduction and 5 percent points in the following years)

•Fast market uptake (15 percent points in the year of introduction and 7,5 percent points in the following years)

•Slow market uptake (5 percent points in the year of introduction and 2,5 percent points in the following years)

Finally, uncertainty was assessed using a probabilistic sensitivity analysis (PSA). The PSA was carried out as a Monte Carlo simulation with 10,000 iterations, simultaneously drawing random numbers for most model inputs from the distributions listed in Table 10 of the Appendix.

The discount rate was set to 0% in all investigated scenarios (Base Case as well as sensitivity analyses), since discounting is – in contrast to cost-effectiveness models – no typical procedure in BIA [73].

## Results

### Base case

According to the results of the Base Case, the introduction of ClosureFast for the treatment of varicose veins saves costs of about 19.1 Mio. € over a time period of five years in Germany. Detailed results are shown in Table 5. Since we assumed equal effectiveness between ClosureFast and surgery, the inpatient and outpatient treatment costs as well as the proportion of inpatient procedures of ClosureFast and surgery, respectively, are the factors which affected the results the most. While the higher outpatient treatment costs of ClosureFast” increases total costs on the one hand, the smaller proportion of inpatient procedures reduces overall costs on the other hand. All in all, the substitution of outpatient for inpatient treatments overcompensate the higher costs of ClosureFast in the outpatient setting. Over a time horizon of five years, a total of 1,623,749 interventional treatments for varicose veins are performed in a “World without ClosureFast”. Of these, about 38% are inpatient procedures causing about 70% of total interventional treatment costs. In a “World with ClosureFast”, according to the results, about 32% of interventional treatments for varicose veins are performed in an inpatient setting causing about 60% of the total interventional treatment costs.

**Table 5:** Results of the base case analysis

	**2013**	**2014**	**2015**	**2016**	**2017**
Total costs (cumulated)*					
World without ClosureFast	402,442,098 €	402,227,395 €	402,175,107 €	402,118,493 €	402,118,493 €
	(402,442,098 €)	(804,669,493 €)	(1,206,844,600 €)	(1,608,963,093 €)	(2,011,081,587 €)
World with ClosureFast	400,534,663 €	399,367,497 €	398,362,261 €	397,352,918 €	396,399,803 €
	(400,534,663 €)	(799,902,160 €)	(1,198,264,421 €)	(1,595,617,339 €)	(1,992,017,142 €)
*Difference*	*−1,907,435 €*	*−2,859,898 €*	*−3,812,846 €*	*−4,765,575 €*	*−5,718,690 €*
	*(−1,907,435 €)*	*(−4,767,333 €)*	*(−8,580,179 €)*	*(−13,345,754 €)*	*(−19,064,445 €)*
**Inpatient costs (cumulated)**					
World without ClosureFast	274,282,077 €	274,162,435 €	274,137,354 €	274,109,746 €	274,109,746 €
	(274,282,077 €)	(548,444,512 €)	(822,581,865 €)	(1,096,691,611 €)	(1,370,801,358 €)
World with ClosureFast	252,637,930 €	241,709,792 €	230,870,333 €	220,030,426 €	209,213,366 €
	(252,637,930 €)	(494,347,722 €)	(725,218,054 €)	(945,248,480 €)	(1,154,461,846 €)
*Difference*	*−21,644,147 €*	*−32,452,643 €*	*−43,267,021 €*	*−54,079,320 €*	*−64,896,380 €*
	*(−21,644,147 €)*	*(−54,096,790 €)*	*(−97,363,811 €)*	*(−151,443,131 €)*	*(−216,339,512 €)*
Outpatient costs (cumulated)**					
World without ClosureFast	119,530,604 €	119,478,465 €	119,467,534 €	119,455,503 €	119,455,503 €
	(119,530,604 €)	(239,009,068 €)	(358,476,603 €)	(477,932,106 €)	(597,387,609 €)
World with ClosureFast	139,267,316 €	149,071,210 €	158,921,709 €	168,769,248 €	178,633,193 €
	(139,267,316 €)	(288,338,526 €)	(447,260,235 €)	(616,029,483 €)	(794,662,676 €)
*Difference*	*19,736,712 €*	*29,592,745 €*	*39,454,175 €*	*49,313,744.85 €*	*59,177,690 €*
	*(19,736,712 €)*	*(49,329,458 €)*	*(88,783,632 €)*	*(138,097,377 €)*	*(197,275,067 €)*
**Inpatient costs/outpatient costs ratio (of cumulated costs)**					
World without ClosureFast	2.29	2.29	2.29	2.29	2.29
	(2.29)	(2.29)	(2.29)	(2.29)	(2.29)
World with ClosureFast	1.81	1.62	1.45	1.30	1.17
	(1.81)	(1.71)	(1.62)	(1.53)	(1.45)

* Including costs of conservative treatment.

** Excluding costs of conservative treatment.

### One-way sensitivity analysis

Results of the one-way sensitivity analyses show that the model outcome is highly sensitive to variations in interventional treatment prices (inpatient as well as outpatient) and the proportion of outpatient treatments. For instance, a 10% increase in the ClosureFast outpatient price leads to additional costs of about 32 Mio € in a “World with ClosureFast”. On the other hand, a 10% price reduction of the ClosureFast outpatient treatment results in additional savings of the same amount. Variations in the values of other input parameters (monthly probability of recurrence, probability of requiring top-up treatment and probability of receiving a 2nd interventional treatment) only had minor effects on the model outcome. Results of all performed one-way sensitivity analyses are shown in Table 11 of the Appendix.

### Scenario analyses

The results of the scenario analyses are presented in Table 6. As one might expect, the introduction of ClosureFast saves more costs in the scenarios with input values favoring a “World with ClosureFast” compared with the Base Case. However, in the two other scenarios (ClosureFast-, ClosureFast--), the introduction of ClosureFast is not cost saving anymore and causes up to 300 Mio € of additional costs for the SHI in five years. A variation of the market uptake does not change the results qualitatively; solely the total costs as well as the cost difference vary between a “World with ClosureFast” and a “World without ClosureFast”.

**Table 6:** Results of the scenario analyses after 5 years

	**Base case**	**ClosureFast+**	**ClosureFast++**	**ClosureFast-**	**ClosureFast--**
**Base Case market uptake of ClosureFast (10 percent points in the year of introduction and 5 percent points in the following years)**					
World without ClosureFast	2,011,081,587 €	2,116,615,576 €	2,431,191,408 €	1,938,122,196 €	1,588,229,622 €
World with ClosureFast	1,992,017,142 €	2,039,393,961 €	2,244,025,455 €	1,996,246,708 €	1,829,572,902 €
*Difference*	*−19,064,445 €*	*−77,221,615 €*	*−187,165,953 €*	*58,124,512 €*	*241,343,280 €*
**Fast market uptake of ClosureFast (15 percent points in the year of introduction and 7.5 percent points in the following years)**					
World without ClosureFast	2,011,081,587 €	2,116,615,576 €	2,431,191,408 €	1,938,122,196 €	1,588,229,622 €
World with ClosureFast	1,982,484,920 €	2,001,065,612 €	2,152,055,284 €	2,025,308,963 €	1,913,810,466 €
*Difference*	*−28,596,667 €*	*−115,549,964 €*	*−279,136,124 €*	*87,186,767 €*	*325,580,844 €*
**Slow market uptake of ClosureFast (5 percent points in the year of introduction and 2.5 percent points in the following years)**					
World without ClosureFast	2,011,081,587 €	2,116,615,576 €	2,431,191,408 €	1,938,122,196 €	1,588,229,622 €
World with ClosureFast	2,001,549,364 €	2,077,910,616 €	2,337,070,830 €	1,967,184,452 €	1,743,326,614 €
*Difference*	*−9,532,223 €*	*−38,704,960 €*	*−94,120,578 €*	*29,062,256 €*	*155,096,992 €*

### Probabilistic sensitivity analysis (PSA)

**Figure 2:**
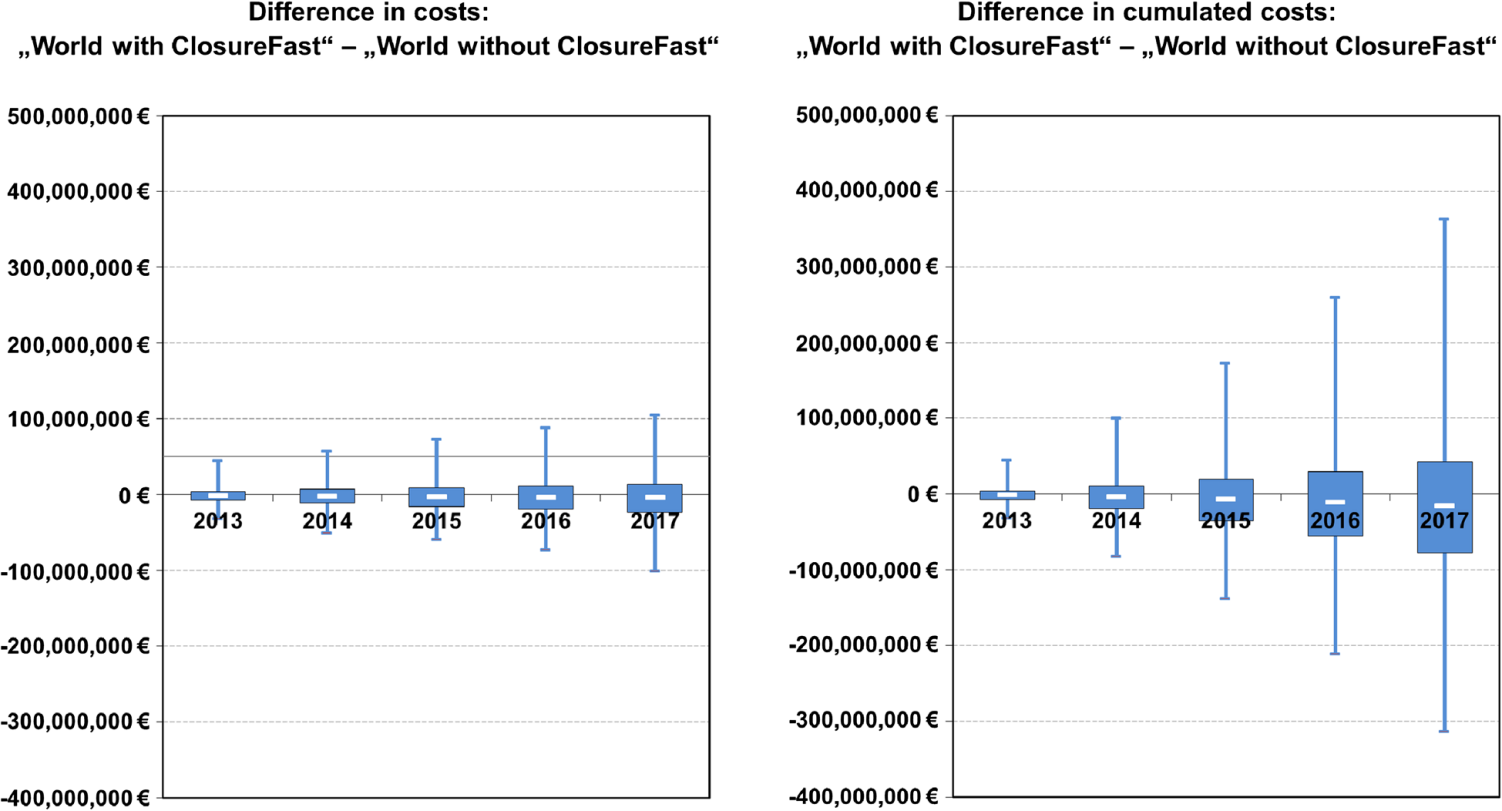
Results of the probabilistic sensitivity analysis.

According to the results of the PSA, illustrated in Figure 2, a “World with ClosureFast” saves costs with a probability of about 59% over a time horizon of five years. With a probability of 25% cost savings of over 75 Mio € can be realized in a “World with ClosureFast”. However, with the same probability (25%) the introduction of ClosureFast is likely to cause additional costs of at least 37 Mio € in the SHI. All in all, it has to be noted that there is high uncertainty regarding the results, which reflects the limited evidence of several key input parameters.

## Discussion

This study is the first to analyze the budget impact of different treatment scenarios for patients with varicose veins in the German setting. According to the results, ClosureFast has the potential to be cost saving for the German SHI.

Our model calculated about 308,000 surgeries of varicose veins in the SHI per year. These figures are based on a combination of the annual number of varicose vein surgeries performed in an inpatient setting and expert opinion. Nüllen et al. estimated that over 350,000 surgery treatments for varices are performed every year in Germany, including private insured patients [74]. Since about 85% of the German population is covered by the SHI [48], the model calculation is in line with the estimation of Nüllen et al. Furthermore, based on the recurrence rates used in the Base Case, our model calculated 248,200 initial surgery procedures which accounts for a proportion of 80.5 percent of all surgery procedures. These results agree with findings in the literature that recurrence treatments account for about 20% [49-53] of all surgery treatments for varicose veins.

In the Base Case scenario, ClosureFast is dominant compared to surgery, since it is cost-saving while being equally effective. This is a conservative estimation, since other studies report a higher effectiveness of ClosureFast [33]. Our findings are supported by the results of the NICE model which also reported dominance of ClosureFast [44]. To our knowledge, there is only one other BIA [75] (for the Ontario setting) which analyses the budget impact of radiofrequency ablation. In contrast to our Base Case analysis, the authors conclude that the introduction of radiofrequency ablation leads to additional costs [75]. A cost-effectiveness study, conducted by Gohel et al., estimated less costs for RFA compared to stripping but also inferior outcomes [76]. In contrast to our analysis, Gohel et al. used effectiveness data of ClosurePlus for their model and only took recurrences over a time horizon of three month into account [76].

The evaluation was conducted from the perspective of the SHI in Germany. Hence, indirect costs of work loss were not included in the analysis. Considering the shorter time to resume work after RFA treatment compared with surgery [18,32,62], cost savings of introducing ClosureFast into the general benefit catalogue of the SHI should be even higher from a social perspective.

This study has some limitations, especially due to a lack of solid data in the literature. The lack of evidence of several key input parameters (in particular expert opinion for the treatment setting) resulted in high uncertainty regarding the outcomes of the model. In addition, there is only limited evidence regarding the effectiveness of treatments for varicose veins in preventing recurrences in the short term and particularly in the long term. Randomized RCTs with high number of patients are missing. Due to the insufficient power, the RCTs [18,36,64,65], which were conducted in this indication could not show any statistical differences in recurrence rates between different RFA and surgery. Therefore, there is urgent need of high quality RCTs.

In general, due to the specialty of the reimbursement system in Germany, the results of this model are not directly transferable to foreign settings. Furthermore, as described earlier, the treatment strategies differ from foreign settings. For instance, surgery is still the dominant treatment strategy in Germany and EVLA plays no role. However, the model structure allows for incorporating EVLA into the analysis and can be adapted to other settings.

## Conclusions

The analysis suggests that the introduction of ClosureFast for patients with varicose veins is cost-saving compared to the status quo in the German SHI setting. Even though the procedure ClosureFast is more expensive in an outpatient setting, cost savings occur due to a substitution of outpatient for inpatient treatments. However, the results scatter in the sensitivity analyses due to limited evidence of some key input parameters.

## Ethical approval for research

Ethical approval is not needed for budget impact analyses.

## Appendix

The files in the appendix provide additional information on reported recurrence rates of varicose veins after interventional treatment (Table 7), the development of SHI prices of interventional treatments for varicose veins (Table 8), a detailed summary and description of all relevant input parameters for the scenario (Table 9) and the probabilistic sensitivity analysis (Table 10) as well as the results of the one-way sensitivity analyses (Table 11).

**Table 7:** Recurrence rates of relevant treatment options reported in RCTs

**Author**	**Year**	**Length of follow-up in months (number of patients at last follow up)**	**Number of patients (limbs)**	**Recurrence rate**
**ClosureFast vs. Surgery vs. UGFS**				
Rasmussen [18]	2011	12 (ClosureFast: 106 Surgery: 97 UGFS: 107)	ClosureFast: 125 (148)	ClosureFast: 9/124 = 7.26%
			Surgery: 125 (143)	Surgery: 16/108 = 14.81%
			UGFS: 125 (145)	UGFS: 17/123 = 13.82%
				Odds ratio (ClosureFastvs Surgery): 0.45
				Odds ratio (ClosureFastvs UGFS): 0.49
				Odds ratio (UGFS vs Surgery): 0.92
**ClosurePlus vs. Surgery**				
Perälä [36]	2005	36 (ClosurePlus: 15 Surgery: 13)	ClosurePlus: 15 (15)	ClosurePlus: 5/15 = 33.33%
			Surgery: 13 (13)	Surgery: 3/13 = 23.1%
				Odds ratio: 1.67
Lurie [64]	2005	24 (ClosurePlus: 36 Surgery: 29)	ClosurePlus: 43 (44)	ClosurePlus: 5/36 = 13.89%
			Surgery: 36 (36)	Surgery: 6/29 = 20.69%
				Odds ratio: 0.62
Helmy Elkaffes [65]	2011	24 (ClosurePlus: 81 Surgery: 81)	ClosurePlus: 90 (90)	ClosurePlus: 12/81 = 14.81%
			Surgery: 90 (90)	Surgery: 9/81 = 11.11%
				Odds ratio: 1.39
**UGFS vs. Surgery**				
Shadid [66]	2012	24 (UGFS: 213 Surgery: 177)	UGFS: 230 (230)	UGFS: 24/213 = 11.27%
			Surgery: 200 (200)	Surgery: 16/177 = 9.04%
				Odds ratio: 1.28
**Foam Sclerotherapy vs Surgery**				
Belcaro [63]	2000	120	Foam Sclerotherapy: 148	Foam Sclerotherapy: 56/148 = 37.84%
			Surgery: 155	Surgery: 38/155 = 24,52%
			Surgery: 200 (200)	Odds Ratio: 1.87

**Table 8:** Development of SHI prices of interventional treatments for varicose veins

	**Surgery**		UGFS (outpatient only)**
	Inpatient*	Outpatient**	
2004	1,811.71 €	n/s	n/s
2005	1,726.92 €	n/s	n/s
2006	1,745.41 €	n/s	n/s
2007	1,821.26 €	n/s	n/s
2008	1,861.95 €	n/s	n/s
2009	2,039.82 €	638.59 €	56.35 €
2010	2,066.19 €	639.45 €	56.43 €
2011	2,074.22 €	639.45 €	56.43 €
2012	2,218.02 €	639.45 €	56.43 €
2013	n/s	645.20 €	56.93 €

* InEK GmbH – Institute for the Hospital Remuneration System: Diagnosis Related Group-Catalogue 204–2012. Düsseldorf: Dt. Krankenhaus-Verl.-Ges.; 2003–2011.

** National Association of Statutory Health Insurance Physicians: Uniform Value Scale 2009–2012. http://www.kbv.de.

**Table 9:** **Input parameters modified in the scenario analyses**^**#**^

**Parameter**	**Base case**	**ClosureFast+**	**ClosureFast++**	**ClosureFast-**	**ClosureFast--**
**Monthly probability of recurrence after 1st and 2nd treatment**					
Surgery	0.00833	0.00833	0.01326**	0.00833	0.00490***
ClosureFast	0.00833	0.00583*	0.00630**	0.00833	0.00666***
UGFS	0.00914	0.00914	0.00914	0.00914	0.00914
* Monthly recurrence rate of endovenous thermal ablation reported by NICE [45]; ** monthly recurrence rate based on Rasmussen et al. [18]; *** monthly recurrence rates based on Helmy Elkaffas et al. [65]					
**Surgery outpatient treatment ratio**					
2013	0.600	0.600	0.600	0.600	0.660**
2014	0.600	0.600	0.600	0.615*	0.690**
2015	0.600	0.600	0.600	0.630*	0.720**
2016	0.600	0.600	0.600	0.645*	0.750**
2017	0.600	0.600	0.600	0.660*	0.780**
* outpatient treatment ratio increases by 1.5 percent points per year; ** outpatient treatment ratio 6 percent points higher in 2013 and increases by 3 percent points per year					
Surgery inpatient costs					
2013	2,218.02 €	2,270.54 €*	2.323,06 €**	2,218.02 €	2,218.02 €
2014	2,218.02 €	2,324.30 €*	2.433,07 €**	2,218.02 €	2,218.02 €
2015	2,218.02 €	2,379.34 €*	2.548,30 €**	2,218.02 €	2,218.02 €
2016	2,218.02 €	2,435.68 €*	2.668,98 €**	2,218.02 €	2,218.02 €
2017	2,218.02 €	2,439.35 €*	2.795,37 €**	2,218.02 €	2,218.02 €
* mean annual price inflation rate 2004–2012 (2.37%); ** mean annual price inflation rate 2004–2012 times two					
**Surgery inpatient treatment costs**					
2013	639.45 €	645.20 €*	645.20* €	639.45 €	639.45 €
2014	639.45 €	645.20 €*	645.20* €	639.45 €	639.45 €
2015	639.45 €	645.20 €*	645.20* €	639.45 €	639.45 €
2016	639.45 €	645.20 €*	645.20* €	639.45 €	639.45 €
2017	639.45 €	645.20 €*	645.20* €	639.45 €	639.45 €
* price based on new EBM point value (0.035363 € per point)					
**ClosureFast outpatient treatment ratio**					
2013	0.90	0.90	0.95	0.90	0.85*
2014	0.90	0.90	0.95	0.90	0.85*
2015	0.90	0.90	0.95	0.90	0.85*
2016	0.90	0.90	0.95	0.90	0.85*
2017	0.90	0.90	0.95	0.90	0.85*
* outpatient treatment ration 5 percent points lower than estimated by experts					
**ClosureFastinpatient costs**					
2013	2,218.02 €	2,270.54 €*	2,023.06 €**	2,218.02 €	2,218.02 €
2014	2,218.02 €	2,324.30 €*	2,118.87 €**	2,218.02 €	2,218.02 €
2015	2,218.02 €	2,379.34 €*	2,219.21 €**	2,218.02 €	2,218.02 €
2016	2,218.02 €	2,435.68 €*	2,324.31 €**	2,218.02 €	2,218.02 €
2017	2,218.02 €	2,493.35 €*	2,434.38 €**	2,218.02 €	2,218.02 €
* mean annual price inflation rate 2004–2012 (2,37%); ** mean annual price inflation rate 2004–2012 times two + inpatient costs of ClosureFast 300 € lower than surgery inpatient costs in 2013					
**ClosureFast inpatient costs**					
2013	1,100.00 €	1,000.00 €	900.00 €	1,300.00 €	1,500.00 €
2014	1,100.00 €	1,000.00 €	900.00 €	1,300.00 €	1,500.00 €
2015	1,100.00 €	1,000.00 €	900.00 €	1,300.00 €	1,500.00 €
2016	1,100.00 €	1,000.00 €	900.00 €	1,300.00 €	1,500.00 €
2017	1,100.00 €	1,000.00 €	900.00 €	1,300.00 €	1,500.00 €
Market shares					
2013-2017	Base Case	Base Case	Base Case	Base Case	*
**Volume increase of the market**					
2013-2017	Base Case	Base Case	Base Case	Base Case	**

^#^ BaseCase values were maintained for all other input parameters.

* Base Case; but ClosureFast takes 2.5 percent points of UGFS market shares in the first year.

** Due to the introduction of ClosureFast the volume of the market increases by 0.5 percent points every year.

**Table 10:** Means, standard errors and distributions of input parameters varied in the probabilistic sensitivity analysis

	**Mean**	**Standard error**	**Distribution**
**Initial intervention population**			
2013-2017	261,263	2,500	normal
**Monthly survival probability**			
	0.99944	0.0001	normal
**Monthly probability of recurrence after initial and 2nd interventional treatment**			
Surgery	0.008331	0.00164	beta
ClosureFast	0.008331	0.00176	beta
UGFS	0.009141	0.00105	beta
**Probability of requiring top-up treatment after initial and 2nd interventional treatment**			
Surgery	0.05000	0.00021	uniform
ClosureFast	0.05000	0.00021	uniform
UGFS	0.20000	0.00333	uniform
**Probability of receiving 2nd interventional treatment**			
	0.75000	0.02083	uniform
**Weighted mean of in- and outpatient interventional treatment costs (per procedure)**			
Surgery	1,270.88 €	190.63 €	gamma
ClosureFast	1,211.80 €	181.77 €	gamma
UGFS	56.43 €	8.46 €	gamma
**Monthly costs of compression therapy**			
	11.45 €	1.14 €	gamma
**ClosureFast market uptake (in percent points)**			
2013	0.10000	0.00083	uniform
2014-2017	0.05000	0.00021	uniform

**Table 11:** Results of one-way sensitivity analysis

	**Input values**			**Results (Difference: “World with ClosureFast” - World without ClosureFast”)**		
	**Base case**	**+ 10%**	**- 10%**	**Base case**	**+ 10%**	**- 10%**
**Monthly probability of recurrence after initial and 2**^**nd **^**interventional treatment**						
Surgery	0.00833	0.00916	0.00750	−19,064,444 €	−22,282,157 €	−15,786,834 €
ClosureFast	0.00833	0.00916	0.00750	−19,064,444 €	−16,130,411 €	−22,067,845 €
UGFS	0.00914	0.01006	0.00823	−19,064,444 €	−19,079,598 €	−19,048,601 €
**Probability of requiring top-up treatment after initial and 2nd interventional treatment**						
Surgery	0.05000	0.05500	0.04500	−19,064,444 €	−19,155,492 €	−18,973,397 €
ClosureFast	0.05000	0.05500	0.04500	−19,064,444 €	−18,973,397 €	−19,155,492 €
UGFS	0.20000	0.22000	0.18000	−19,064,444 €	−19,064,444 €	−19,064,444 €
**Probability of receiving 2nd interventional treatment**						
	0.75000	0.82500	0.67500	−19,064,444 €	−19,427,439 €	−18,701,450 €
**Inpatient costs of interventional treatment (per procedure)**						
Surgery	2,218.02 €	2,439.82 €	1,996.22 €	−19,064,444 €	−47,695,356 €	9,566,467 €
ClosureFast	2,218.02 €	2,439.82 €	1,996.22 €	−19,064,444 €	−11,906,717 €	−26,222,172 €
**Outpatient costs of interventional treatment (per procedure)**						
Surgery	639.45 €	703.40 €	575.51 €	−19,064,444 €	−31,445,797 €	−6,683,092 €
ClosureFast	1,100.00 €	1,210.00 €	990.00 €	−19,064,444 €	12,883,647 €	−51,012,536 €
UGFS	56.43 €	62.07 €	50.78 €	−19,064,444 €	−19,064,444 €	−19,064,444 €
**Proportion of outpatient treatments**						
Surgery	0.60000	0.66000	0.54000	−19,064,444 €	11,500,570 €	−49,629,459 €
ClosureFast	0.90000	0.99000	0.81000	−19,064,444 €	−51,535,903 €	13,407,014 €
**Monthly costs of compression therapy**						
	11.45 €	12.59 €	10.30 €	−19,064,444 €	−19,064,444 €	−19,064,444 €

## Abbreviations

BIA: Budget impact analysis; EVLA: Endovenous laser ablation; PSA: Probabilistic sensitivity analysis; RFA: Radiofrequency ablation; UGFS: Ultrasound guided foam sclerotherapy; SHI: Statutory health insurance.

## Competing interests

The study is financially supported by Covidien.

## Authors’ contributions

AK has constructed the Markov model in Microsoft Excel. AK and AP gathered the necessary data as well as analyzed and interpreted the results. AK and AP have also writ-ten the manuscript. JMS and JH have revised the manuscript for important intellectual content. All authors have read and approved the final manuscript.
